# Need for achievement moderates the effect of motive-relevant challenge on salivary cortisol changes

**DOI:** 10.1007/s11031-014-9465-7

**Published:** 2014-12-24

**Authors:** Fang Yang, Jonathan E. Ramsay, Oliver C. Schultheiss, Joyce S. Pang

**Affiliations:** 1 School of Sociology and Political Science, Shanghai University, Shanghai, China; 2 UniSIM College, SIM University, Clementi, Singapore; 3 Department of Psychology, Friedrich-Alexander University, Erlangen, Germany; 4 Division of Psychology, School of Humanities and Social Sciences, Nanyang Technological University, HSS-04-08, 14 Nanyang Drive, Singapore, 637332 Singapore

**Keywords:** Need for achievement, Implicit motives, Cortisol, Stress, HPA axis, Picture story exercise

## Abstract

The hypothalamic–pituitary–adrenal (HPA) axis plays a key role in the physiological response to stress, preparing the organism for appropriate action. While some research has examined universally relevant threats, other research has suggested that individual differences may moderate the relationship between stress and cortisol release, such that some individuals exhibit modified reactivity to personally relevant stressors or challenges. In the present study we investigated whether one individual difference—the implicit need for achievement—moderates the effect of motive-relevant challenge on salivary cortisol. Participants’ salivary cortisol and felt affect were measured before and after engagement in an achievement task. In the positive- and no-feedback conditions, individuals high in implicit achievement motivation demonstrated increased cortisol response to the task, whereas in the negative feedback condition, individuals high in implicit achievement motivation demonstrated a dampened cortisol response. Furthermore, changes in cortisol were accompanied by changes in felt affect in the same direction, specifically hedonic tone. These results suggest that the HPA axis also responds to non-social-evaluative challenge in a personality-contingent manner.

## Implicit achievement motive moderates the effect of motive-relevant challenge on salivary cortisol changes

In humans and animals alike, threats to survival and personal safety are considered basic external stressors, and research suggests that the hypothalamic–pituitary–adrenal (HPA) axis is activated in response to perceptions of mortal danger (Sapolsky et al. 2000), leading to the release of cortisol. However in more evolutionarily advanced organisms, less tangible and less immediate threats are also capable of activating the HPA-stress response. Threats to social standing are extremely effective stressors in highly social mammalian species, and an extensive animal literature has documented increased HPA activity in chronically or acutely threatened individuals (Blanchard et al. 1993). Humans are no exception; hence Dickerson and Kemeny’s (2004) meta-analysis found that social-evaluative stressors, such as the widely used Trier Social Stress Test (TSST; Kirschbaum et al. 1993) induce substantial cortisol release. However, while the effects of social-evaluative stress on cortisol reactivity are well documented, other researchers have also examined the effectiveness of challenging, non-social-evaluative tasks in activating the HPA axis. In their meta-analysis, Dickerson and Kemeny (2004) found that non-social-evaluative motivated performance tasks featuring uncontrollable elements gave rise to effect sizes that differed significantly from zero. In laboratory studies, cognitive tests of perceptual skill (e.g., mirror tracing; Steptoe et al. 2001), vigilance and attention (e.g., Szalma et al. 2004), or mathematical ability under time pressure (e.g. Lejuez et al. 2003) have also been found to be potent stressors, given that they significantly tax an individual’s mental resources. For instance, Agrigoroaei et al. (2013) found that participants with higher perceived control experienced greater cortisol response during a driving simulation challenge with low controllability. While social judgments are among the most effective cortisol-inducing stressors, the ability of non-social-evaluative tasks to activate the HPA response (Dickerson and Kemeny 2004) suggests a more general role for cortisol and the HPA axis in responding to challenging situations.

Furthermore, a body of evidence spanning many diverse literatures supports the assertion that the ability of specific stressor classes to induce HPA activity is moderated by a number of individual difference variables that potentiate the effects of personally relevant challenges. For instance, extraversion (Davis et al. 1999; Oswald et al. 2004), chronic perceptions of sexism (Townsend et al. 2011), callous-unemotional traits (Shirtcliff et al. 2009), and clinical depression and anxiety (Young et al. 2000, 2004) are just a few of the individual difference variables which have been implicated in the modification of the stress response, enhancing or dampening HPA function depending on the personal relevance of the stressor in question.

Despite this evidence, it is likely that many such moderators remain undiscovered, and implicit motives can be considered promising candidates in the continuing search. Implicit motives are fundamental building blocks of personality (Winter et al. 1998): individual differences in the tendency “to be concerned with and to strive for certain classes of incentives or goals”(Emmons 1989, p. 32). They are implicit in the sense that they cannot be validly assessed with self-report measures, but are instead inferred indirectly, frequently via the content-coding of imaginative stories that participants write in response to picture cues. Implicit motives vary considerably among individuals. While some individuals may derive satisfaction primarily through the formation and maintenance of close relationships (the need for affiliation; *n*Aff) or the exertion of influence on social others (the need for power; *n*Pow), others may favor the affective rewards associated with proficiency and mastery of challenging tasks. This need for achievement, defined by Pang (2010b, p. 30) as a “preference for affectively rewarding experiences related to improving one’s performance”, is among the most widely studied of human motives (Weiner 2013), and has been associated with a range of domain-relevant outcomes, such as academic performance (McKeachie et al. 1968), and entrepreneurial success (McClelland 1987).

Previous research suggests that implicit motives can moderate the cortisol response to motive-relevant challenge. Wirth et al. (2006) found that *n*Pow predicted salivary cortisol response to dominance challenges, with high *n*Pow individuals, but not low *n*Pow individuals, demonstrating an increase in cortisol in response to defeat and a decrease in response to victory. This suggests that motivation affects the sensitivity of hormonal systems to motive-relevant events, such as dominance challenges in the case of *n*Pow. If *n*Pow predicts the cortisol response to stressful power-related challenge, then it seems reasonable that *n*Ach might predict HPA activation particularly in response to achievement-related stress. Since implicit motives determine the primary domains in which individuals derive satisfaction from incentives and experience stress and frustration from disincentives (McClelland 1987), moderation of HPA reactivity may be one way in which this domain specificity is expressed.

Implicit motives affect an individual’s construal of situational characteristics. In turn, these construals such as those regarding the novelty, controllability, and predictability of events modulate the degree of stress an individual experiences in the situation, as well as the concomitant cortisol response (Mason 1975; Wirth and Gaffey 2013). One notable instance excepting (which we shall discuss shortly), the potential for *n*Ach to moderate cortisol response to achievement-relevant stress has not been systematically tested, and investigating this possibility is the focus of the present research. Previous work on *n*Ach has revealed that achievement motivated individuals value moderately challenging tasks and perform better at these tasks (Feather 1962; Ramsay and Pang 2013), seek feedback that is diagnostic of their ability and which allows them to improve on their performances (Brunstein and Maier 2005; Fodor and Carver 2000), and are intrinsically motivated to perform a task “for its own sake” (French 1955; McKeachie 1961). In other words, achievement-motivated individuals are predisposed to seek out and prefer being in contexts where they can excel, or improve their performance. These findings suggest that moderately challenging tasks that provide diagnostic performance feedback should be affectively rewarding, and consequently, more manageable and less stressful for *n*Ach-motivated individuals, because these tasks provide the opportunity to obtain mastery. Thus, we expected that differences in cortisol reactivity within achievement motive-relevant conditions might be affected by stable differences in *n*Ach.

To date, we are aware of only one paper that has attempted to explore the moderating role of achievement motivation on cortisol response. Schultheiss et al. (2014) examined the effects of *n*Ach on cortisol reactivity after a dominance contest (study 1) and in response to the Trier Social Stress Task (Kirschbaum et al. 1993; study 2). They found that achievement motivation dampened the cortisol response only after the challenging dominance- and social stress-related tasks, but not in a non-challenging control condition (study 2 only). Thus, their findings suggest that achievement motivation can help to moderate the cortisol response in challenging contexts even when good performance in these contexts is in service of other motives such as power motivation or impression management. Our research extends Schultheiss et al.’s work (2014) in two ways. First, we focus on the effect of *n*Ach on cortisol response during motivationally relevant, achievement-challenge contexts. Schultheiss et al.’s conditions were ostensibly more related to other motives such as *n*Power, which is aroused in competitive situations as well as in persuasive tasks. Specifically, as explained later, we also investigate the finer details of what constitutes *achievement*-*relevant challenge* by examining whether *n*Ach moderates the cortisol response even under different types of performance feedback (positive, negative, or none) received in an achievement setting. Second, both tasks in Schultheiss et al.’s paper could be construed as social-evaluative scenarios, since in both tasks inter-personal interaction was an important component of good performance. In our research, we use a task for which good performance does not rely on interpersonal interactions. Thus, we are able to examine whether motives modulate the cortisol response to motive-relevant challenge, even when the challenge/stress occurs during non-socially evaluative settings.

### Cortisol change and affect change

In addition, we explored the effect of *n*Ach on affective response to an achievement-relevant challenging task, which represents another contribution to the literature. Specifically, in this study, we explored whether motive-based differential cortisol responses to a challenging task are accompanied by parallel changes in felt affect. In keeping with Atkinson’s (1957) view of implicit motives as affect amplifiers, we view affect as inextricably linked to implicit motives, signaling the presence of relevant incentives within the environment in order to modify behavior and thus increase the likelihood of reward. Furthermore, implicit motives predispose people to pursue accomplishments in motive-congruent domains (e.g., challenging tasks requiring effort and skill in the case of *n*Ach), in order to obtain the pleasure that accompanies success (Atkinson 1957). In other words, those high in *n*Ach are more likely to experience positive affect while engaging in challenging tasks than those low in *n*Ach. Thus, changes in felt affect may be another indicator, besides HPA activation and/or inhibition, of the presence of achievement-specific incentives.

### The present research

#### The moderating role of nAch

The present study investigated the potential moderating effect of *n*Ach on the relationship between achievement-related challenge and HPA activation. Specifically, we sought to ascertain whether *n*Ach moderated the effects of participation in a demanding cognitive task on salivary cortisol. Given our characterization of *n*Ach as an affectively-based preference for seeking competence in achievement settings, we further sought to investigate whether the moderating effect of *n*Ach on the challenge-cortisol relationship was accompanied by changes in felt affect. Our specific hypotheses were as follows: Given that achievement-motivated individuals perceive challenging tasks as greater opportunities to experience the positive affect associated with proficiency and mastery (Schultheiss and Brunstein 2005), we hypothesized that individuals high in *n*Ach may view the challenging task as less stressful and thus would display a lower cortisol response to motive-relevant cognitive challenge compared to those lower in *n*Ach (H1). Such results would be consistent with the findings of Schultheiss et al. (2014), who demonstrated such an attenuated cortisol response to challenge in *n*Ach-motivated individuals in less motivationally relevant settings. Furthermore, it is well-documented that individuals high in *n*Ach enjoy challenging tasks more than those low in *n*Ach (e.g., Reeve et al. 1987). As such, we hypothesized that individuals with high *n*Ach would display greater positive change in felt affect in response to cognitive challenge than low *n*Ach individuals (H2).

#### The role of performance feedback

Besides examining whether *n*Ach moderates the relationship between achievement-related challenge and cortisol reactivity and felt affect, we also explored whether these hypothesized *n*Ach-dependent changes in cortisol and felt affect would depend on the type of performance feedback they received. Previous research has demonstrated that self-referenced feedback in challenging tasks arouses *n*Ach (e.g., Brunstein and Maier 2005; Brunstein and Schmitt 2004), yet the question of whether positive or negative feedback is more potent remains unresolved. Some researchers have theorized that achievement-motivated individuals should prefer positive feedback to negative feedback (e.g., Halisch and Heckhausen 1989), whereas other researchers (e.g., Trope 1975) have suggested that the valence of the feedback matters less for achievement motivated individuals than the diagnostic potential of the feedback for distinguishing between degrees of competence. Given this uncertainty, we refrained from making specific hypotheses regarding the differential effects of positive and negative feedback. However, since previous research (e.g., Halisch and Heckhausen 1989; McClelland et al. 1953; Ramsay and Pang 2013) has generally shown that mixed-valence performance feedback is an effective incentive for *n*Ach motivated individuals, we suspected that the positive and negative feedback conditions would be more intrinsically rewarding for achievement motivated individuals, and would therefore be more likely to reveal the hypothesized moderation effect of *n*Ach on cortisol reactivity, compared to a no-feedback condition.

## Methods

### Participants

Fifty participants (26 females, *M*_age_ = 18.77 years, *SD* = 0.95; 24 males, *M*_age_ = 19.92 years, *SD* = 2.98) were recruited through the University of Michigan introductory psychology subject pool, as well as through flyers advertising a paid research study open to undergraduate and graduate students not majoring in psychology. Participants from the subject pool received 1 h of academic credit, while participants recruited via flyers received $10 per hour as recompense for their participation. Use of hormonal contraceptives was an exclusionary criterion, while three participants were further excluded from the final analysis as they did not provide either implicit motive or affect data.

### Procedure

The experimental sessions lasted for approximately 1 h and took place between 11:00 and 18:00. Data were collected over a period of 5 months between February and June 2004. At the beginning of the session, participants completed a picture-story exercise that took about 20 min. Immediately afterwards, they completed an affect questionnaire and provided a saliva sample for cortisol assessment (T1). Participants then completed a version of the d2 test of attention that lasted around 15 min, after which participants completed a second version of the earlier affect questionnaire and several other measures that are not the focus of the present analysis, before providing another saliva sample for cortisol assessment (T2). As such, there was a delay of approximately half an hour between completion of the cognitive task and the second cortisol measurement. All tasks and measures were administered via computer using Experimental Run Time System (Berisoft Corporation, Frankfurt am Main, Germany).

### Experimental task

**Fig. 1:**
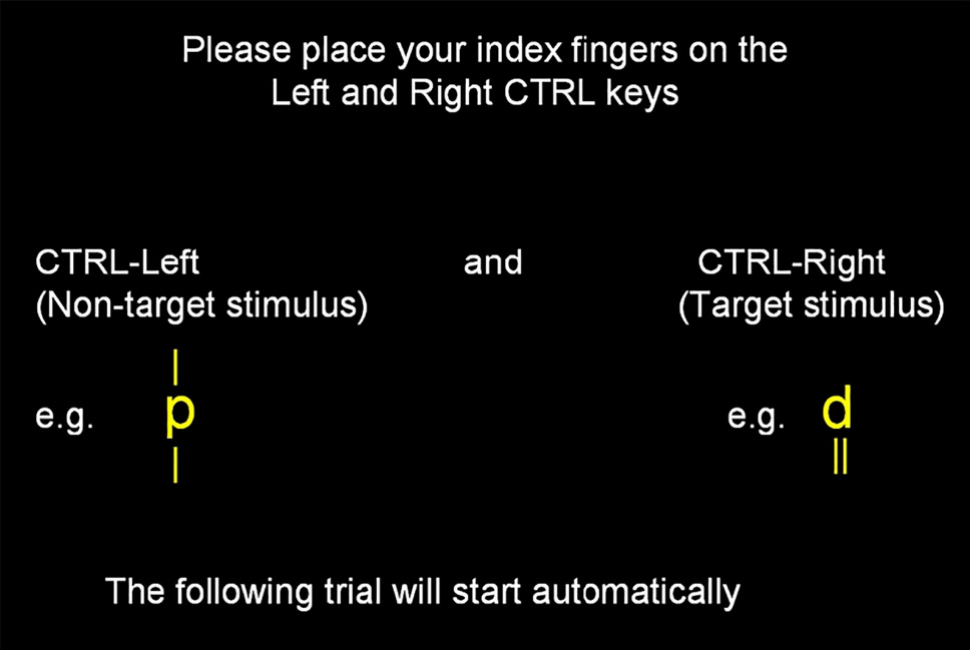
Instruction screen in the d2 test of attention

The d2 test of attention (Brickenkamp and Zillmer 1998) is a widely used measure of selective attention and a commonly used challenging cognitive task (Goldhammer et al. 2007). We used a modified version of the d2 test to provide achievement-relevant challenge in the present study, as a great deal of mental effort is needed to perform effectively on this task. The d2 test requires participants to identify specific visual stimuli under time pressure, and has been used extensively in research examining the interplay of performance feedback and implicit achievement motivation (e.g. Brunstein and Gollwitzer 1996; Brunstein and Hoyer 2002; Brunstein and Maier 2005; Kuhl 1981). Generally, individuals completing the d2 test have described it as being highly involving, which indicates a task of potent challenge (Brunstein and Gollwitzer 1996). In our version of the task, the letters “p” and “d” were presented with either one or two dashes, arranged either individually or in pairs, above or below the letter. Participants were asked to respond as quickly and accurately as possible, pressing the right CTRL key on their keyboard whenever they saw the target stimulus (a letter “d” presented with two dashes) and pressing the left CTRL key whenever non-target stimuli were encountered (see Fig. 1). The instruction that encourages participants to respond as quickly and accurately as possible and to demonstrate their competence could potentially facilitate their engagement level (Brunstein and Gollwitzer 1996). Participants first completed a series of 120 training trials (arranged into two blocks of 60 trials each) during which no feedback was provided. Once the training trials were complete, participants were told that their subsequent performance in the experimental trials would be monitored by the computer, and that they would be provided with feedback based on their performance during the training trials. Participants then proceeded to complete the main experimental trials, which were arranged into ten blocks of 18 trials each. The average reaction time was 556 ms (*SD* = 57), and the average accuracy rate was 46 % (*SD* = 0.33), which suggests that the task is quite challenging for our participants.

The nature of the feedback provided during the experimental trials varied among participants. Participants were randomly assigned to one of three conditions: positive feedback (*N* = 19), negative feedback (*N* = 15), or no feedback (*N* = 13). Contrary to the instructions, all feedback was manipulated and had no actual relationship to performance on the practice trials. In the positive feedback condition, participants were given positive performance feedback (presentation of a schematic happy face, accompanied by the phrase “you performed significantly better than during the training trials”) at the end of blocks 2, 3, 7, and 9 of ten total experimental blocks. In the negative feedback condition, participants were presented a schematic unhappy face, accompanied by the phrase “you performed significantly worse than during the training trials” at the end of experimental blocks 2, 3, 7, and 9. In both feedback conditions, participants received a blank screen after blocks 1, 4, 5, 6, 8, and 10. Participants in the no feedback condition received no feedback on their performance at all. Instead they received a blank screen at the end of each block.

### Picture-story exercise

*n*Ach was measured using the picture story exercise (PSE; Pang and Schultheiss 2005). In the PSE, participants are shown picture cues portraying actors in ambiguous situations. In response to each image, participants are required to write an imaginative story describing what is happening in the picture. In the present study, participants were presented with four images, and were given 4 min to write each story. The pictures used were chemist (Pang et al. 2009), piano lesson (Ramsay and Pang 2013), mechanics (Pang 2006) and soccer duel (Pang 2010a). Stories were analyzed using Pang’s (2006) revised manual for hope of success (HS), which provided a measure of *n*Ach. Pang’s HS was chosen on the grounds that it focuses purely on the approach-related aspects of achievement motivation, unlike other commonly-used coding systems such as McClelland et al. (1953) and Winter (1994) which collapse approach and avoidance-related components into a single measure, a practice that leads to difficulties in the interpretation of results. The Pang (2006) system specifies eight categories of approach-related achievement imagery which are combined to provide a measure of HS: an individual’s motivation to succeed. Despite their similarities, Pang’s (2006) coding system was preferred to Heckhausen’s (1963) system on the grounds that its constituent coding categories are empirically rather than theoretically derived, a quality that may increase construct validity (see Pang 2010b, for a discussion). The Pang system displays convergent validity with Heckhausen system (Pang and Ramsay 2013) and has also been shown to exhibit predictive validity in several behavioral domains (Pang and Ramsay 2013; Pang 2010a). Two independent coders coded the stories with an intra-class coefficient of greater than .70 (Meyer et al. 2002), and the final motive scores were averaged between coders and corrected for word count using a method recommended by Schultheiss and Pang (2007), in which the raw motive scores are divided by the number of words then multiplied by 1,000 [word count: *M* = 392.94, *SD* = 75.93; *n*Ach (word count corrected): *M* = 2.32, *SD* = 1.80; correlation between the two: *r* = .01, *ns*].

### Saliva collection and hormone assays

At each sampling point, participants used a fresh sugar-free chewing gum to stimulate saliva flow in order to obtain 2 ml saliva in a sterile polypropylene vial. Samples were freed from mucopolysaccarides and other residuals by three freeze–thaw cycles with subsequent centrifugation for 15 min at 4 °C and 1,500 g. Salivary cortisol levels were determined by solid-phase 125I radioimmunoassays, using commercially available kits (DPC Coat-A-Count, now available through Siemens Healthcare Diagnostics, Duluth, GA). Mean intra-assay coefficient of variation (CV) for all participant samples was 6.54 %, and mean interassay CV across 2 saliva pools was 10 %. Mean assay sensitivity (B0-3 SD) was 0.019 ng/ml. Recovery for a low (1.5 ng/ml) and a high (3.5 ng/ml) sample was 109 and 106 % respectively.

### Mood adjective checklist

Participants’ felt affect was measured by the UWIST Mood Adjective Check List (UMACL; Matthews et al. 1990). The UMACL comprises a list of 24 adjectives, which participants are required to rate on a four-point scale from *definitely* to *definitely not*, depending on how well the adjective describes their present affect. The UMACL comprises three independent scales—energetic arousal (e.g., energetic, sluggish), tense arousal (e.g. nervous, calm), and hedonic tone (e.g., satisfied, depressed)—each of which is measured by eight items. The minimum score on each subscale is eight and the maximum is 32. Reliabilities for these three scales were .81, .80, .85 (T1), and .82, .80, .81 (T2) respectively.

### Statistical analysis

The analyses were conducted with IBM SPSS 21 and SYSTAT 12 and involved *t* test, bivariate correlation, bi-partial correlation analysis and ANCOVA. During the course of these analyses, it was found that one participant in the negative feedback condition had a *n*Ach score that was more than 3 standard deviations (3.56 SD) above the group mean (hereafter referred to as Participant A). Participant A’s *n*Ach score would be considered a case with high leverage from the traditional statistical point of view, and might therefore warrant exclusion from the final analysis. However, recent discussions of best practices in psychological research (c.f., Funder et al. 2014) have called for greater transparency in reporting of data and results, and has cautioned against unwarranted exclusion of data points: a questionable research practice that may misrepresent results. With reference to the outlier handling advice provided by Cohen et al. (2003), we did not find evidence of errors in execution, measurement, recording, or aggregation that would justify the exclusion of this case. Nevertheless, in response to a reviewer’s request, we present both sets of results (including and excluding Participant A’s data) in the section below.

## Results

### Results including participant A

#### Preliminary analysis

In order to investigate possible gender differences, we first conducted independent samples *t* tests to compare mean male and female levels of *n*Ach, cortisol (both pre- and post-test), and felt affect (both pre- and post-test). We found no significant gender differences in any of these variables. As such, the remaining analyses were performed on the entire sample. Descriptive statistics are shown in Table 1.

**Table 1:** Descriptive statistics and correlations of variables in the study

	1	2	3	4	5	6	7	8	9
1. *n*Ach	1								
2. Pre-task C	−.12	1							
3. Post-task C	.04	.83^***^	1						
4. Pre-task EA	.00	.09	.21	1					
5. Post-task EA	.19	−.11	.08	.64^***^	1				
6. Pre-task TA	.12	−.11	.01	−.27	−.10	1			
7. Post-task TA	−.14	−.05	.03	−.13	−.15	.75^***^	1		
8. Pre-task HT	−.02	.12	.05	.64^***^	.36^*^	−.58^***^	−.34^*^	1	
9. Post-task HT	.24	.12	.21	.59^***^	.61^***^	−.46^***^	−.52^***^	.70^***^	1
M	6.23	1.03	0.78	19.66	19.23	15.70	16.57	23.68	23.36
SD	5.01	0.46	0.40	4.08	4.64	4.59	4.12	4.81	4.50

*nAch* = need for achievement, word count corrected scores (the raw motive scores are divided by the number of words then multiplied by 1,000)

*C* cortisol (log-transformed), *EA* energetic arousal, *TA* tense arousal, *HT* hedonic tone

*** *p* < .001; * *p* < .05

#### ANCOVA and follow-up analysis

We used ANCOVA to investigate the effect of *n*Ach on post-task cortisol, with cortisol log-transformed while statistically accounting for baseline cortisol. A total of four models were tested, each with a different number of predictors. Model 1 was the simplest, including only baseline cortisol, while Model 4 was the most complex, including *n*Ach and baseline cortisol as well as feedback condition and terms representing the interaction of *n*Ach and feedback with no feedback as the reference condition. Results of these analyses can be found in Table 2. In each of the four models, pre-task cortisol was found to be a highly significant predictor of post-task cortisol. *n*Ach did not significantly predict post-task cortisol in Models 2 and 3. In Model 4, *n*Ach was found to be significant, and the interaction of *n*Ach and negative feedback significantly predicted post-task cortisol, while the interaction of *n*Ach and positive feedback was not significant when no feedback was used as the reference category.

**Table 2:** ANCOVA results for post-task cortisol (*N* = 47)

Variable	Model 1				Model 2				Model 3				Model 4			
	*B*	*SE*	*t*	*p*	*B*	*SE*	*t*	*p*	*B*	*SE*	*t*	*p*	*B*	*SE*	*t*	*p*
Pre-task cortisol^a^	0.72	0.07	10.13	.000	0.74	0.07	10.49	.000	0.73	0.07	10.03	.000	0.72	0.06	11.30	.000
*n*Ach^b^					0.01	0.01	1.73	.090	0.01	0.01	1.78	.082	0.03	0.01	2.58	.014
Positive FB									−0.03	0.08	−0.33	.744	−0.04	0.07	−0.58	.563
Negative FB									−0.06	0.09	−0.64	.524	−0.05	0.08	−0.61	.546
*nAch × positive*																
FB^c^													0.00	0.02	−0.16	.877
*nAch × negative*																
FB^c^													−0.05	0.02	−3.07	.004
*R* ^2^	.695				.715				.717				.800			
∆*R* ^2^					.019				.003				.083^***^			

*nAch* need for achievement, *FB* feedback

*** *p* < .001; ** *p* < .01

^a^Log-transformed

^b^Word corrected and mean-centered

^c^Reference category is no feedback condition

Parallel ANCOVA analyses were also run to test the effect of *n*Ach on the affect variables. We found a significant main effect of *n*Ach on post-task tense arousal (*B* = −0.20, *SE* = 0.08, β = −.24, *p* = .014) and on post-task hedonic tone (*B* = 0.23, *SE* = 0.09, β = .25, *p* = .017), but no significant main effect on energetic arousal nor significant interactions between *n*Ach and condition on the affect variables.

**Fig. 2:**
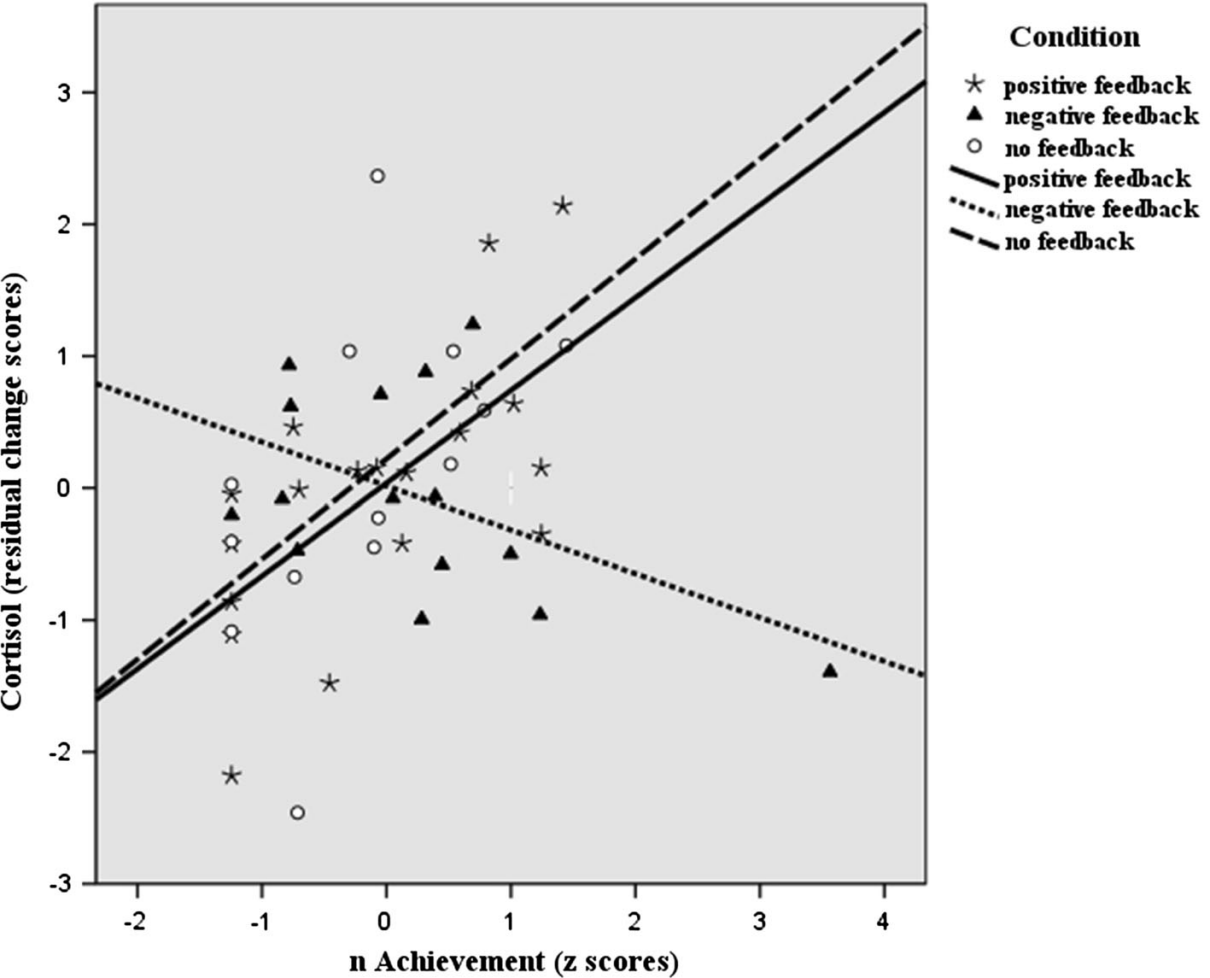
Relationship between *n*Ach and residualized cortisol scores in the three experimental conditions (N = 47)

**Fig. 3:**
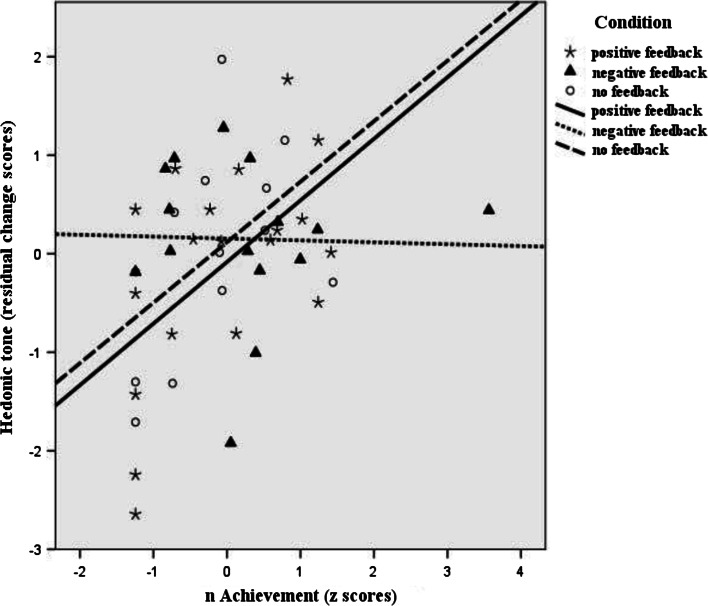
Relationship between *n*Ach and residualized hedonic tone scores in the three experimental conditions. (N = 47)

Furthermore, we examined the relationships between *n*Ach and residualized post-task cortisol and felt affect first in the whole sample and subsequently in each condition separately as follow-up analysis (Table 3). Results of the whole sample analysis showed that *n*Ach was significantly related to lower tense arousal (*r* = −.36, *p* = .014), and higher hedonic tone (*r* = .35, *p* = .015), but was not significantly related to residualized post-task cortisol or energetic arousal. Analyzing the feedback conditions separately, we found that *n*Ach was significantly related to both higher residualized post-task cortisol (*r* = .66, *p* = .002) and hedonic tone (*r* = .54, *p* = .016) in the positive feedback condition, but not significantly related to energetic arousal and tense arousal. Marginally significant correlations were found between *n*Ach and lower residualized post-task cortisol (*r* = −.50, *p* = .061) in the negative feedback condition, and between *n*Ach and higher residualized post-task cortisol (*r* = .54, *p* = .060) in the no feedback condition. We illustrated these results as scatterplots in Figs. 2, 3.

**Table 3:** Bivariate correlation between Z score of *n*Ach and residualized outcomes (*N* = 47)

Condition		Residualized cortisol	Residualized EA	Residualized TA	Residualized HT
The whole sample	*n*Ach	.25	.25	−.36^*^	.35^*^
Positive feedback (N = 19)	*n*Ach	.66^**^	.21	−.31	.54^*^
Negative feedback (N = 15)	*n*Ach	−.50^+^	.20	−.33	−.03
No feedback (N = 13)	*n*Ach	.54^+^	.30	−.52	.50

*nAch* need for achievement, *EA* energetic arousal, *TA* tense arousal, *HT* hedonic tone

** *p* < .01; * *p* < .05; + *p* < .07

#### Cortisol change and affect change

Utilizing the entire sample, bi-partial correlation analysis was performed to examine whether changes in cortisol co-varied with any of the affective variables. Results showed that the pre- to post-task change in hedonic tone was positively related to the change in cortisol, *F* (1, 44) = 7.422, *p* = .009, bipartial *r* = .38. However, no significant correlations were found between the change in cortisol and the changes in either energetic or tense arousal.

### Results excluding participant A

Participant A (*n*Ach = 3.56 *SD*) was excluded, and the remaining analyses were conducted utilizing the same statistical approach as above. For the sake of parsimony, we will not report the similar results in detail. Instead, we highlight the differences between the two results.

#### ANCOVA and follow-up analysis

Similarly, results revealed no gender differences in the key variables of the study, and the analyses were performed on the whole sample.

ANCOVA results for post-task cortisol show that pre-task cortisol was a highly significant predictor of post-task cortisol across the four models. In Model 4, we found the interaction between *n*Ach and negative feedback significant, but not for the interaction between *n*Ach and positive feedback, when no feedback was used as the reference category (Table 4). The results differ from the findings above in that *n*Ach was significant in Models 2 and 3.

**Table 4:** ANCOVA results for post-task cortisol (*N* = 46)

Variable	Model 1				Model 2				Model 3				Model 4			
	*B*	*SE*	*t*	*p*	*B*	*SE*	*t*	*p*	*B*	*SE*	*t*	*p*	*B*	*SE*	*t*	*p*
Pre-task cortisol^a^	0.71	0.07	10.04	.000	0.73	0.07	11.25	.000	0.73	0.07	10.82	.000	0.72	0.06	11.19	.000
*n*Ach^b^					0.02	0.01	3.19	.003	0.02	0.01	3.15	0.003	0.03	0.01	2.55	.015
Positive FB									−0.03	0.07	−0.45	0.657	−0.04	0.07	−0.58	.567
Negative FB									−0.03	0.08	−0.39	0.701	−0.05	0.08	−0.55	.583
*nAch × positive*																
FB^c^													0.00	0.02	−0.16	.879
*nAch × negative*																
FB^c^													−0.04	0.02	−2.26	.030
*R* ^2^	.696				.754				.755				.792			
∆*R* ^2^					.058^**^				.001				.037^*^			

*nAch* need for achievement, *FB* feedback

*** *p* < .001; ** *p* < .01

^a^Log-transformed

^b^Word corrected and mean-centered

^c^Reference category is no feedback condition

Similar ANCOVA results for the affect variables were found. Results show a significant main effect of *n*Ach on post-task tense arousal (*B* = −0.23, *SE* = 0.09, β = −.24, *p* = .014) and on post-task hedonic tone (*B* = 0.28, *SE* = 0.11, β = .27, *p* = .011), but no significant main effect on energetic arousal nor significant interactions between *n*Ach and condition on the affect variables.

**Fig. 4:**
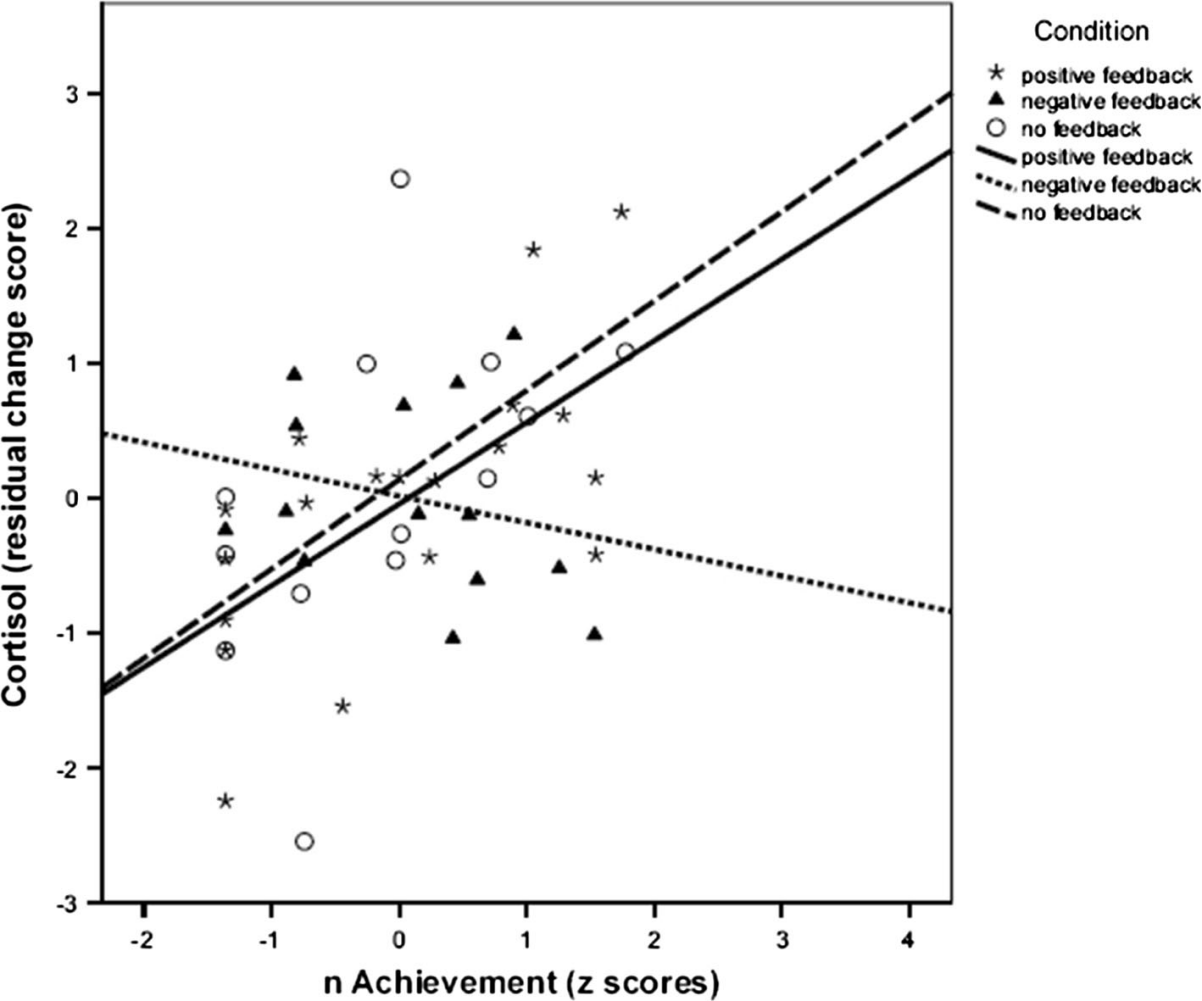
Relationship between *n*Ach and residualized cortisol scores in the three experimental conditions. (N = 46)

**Fig. 5:**
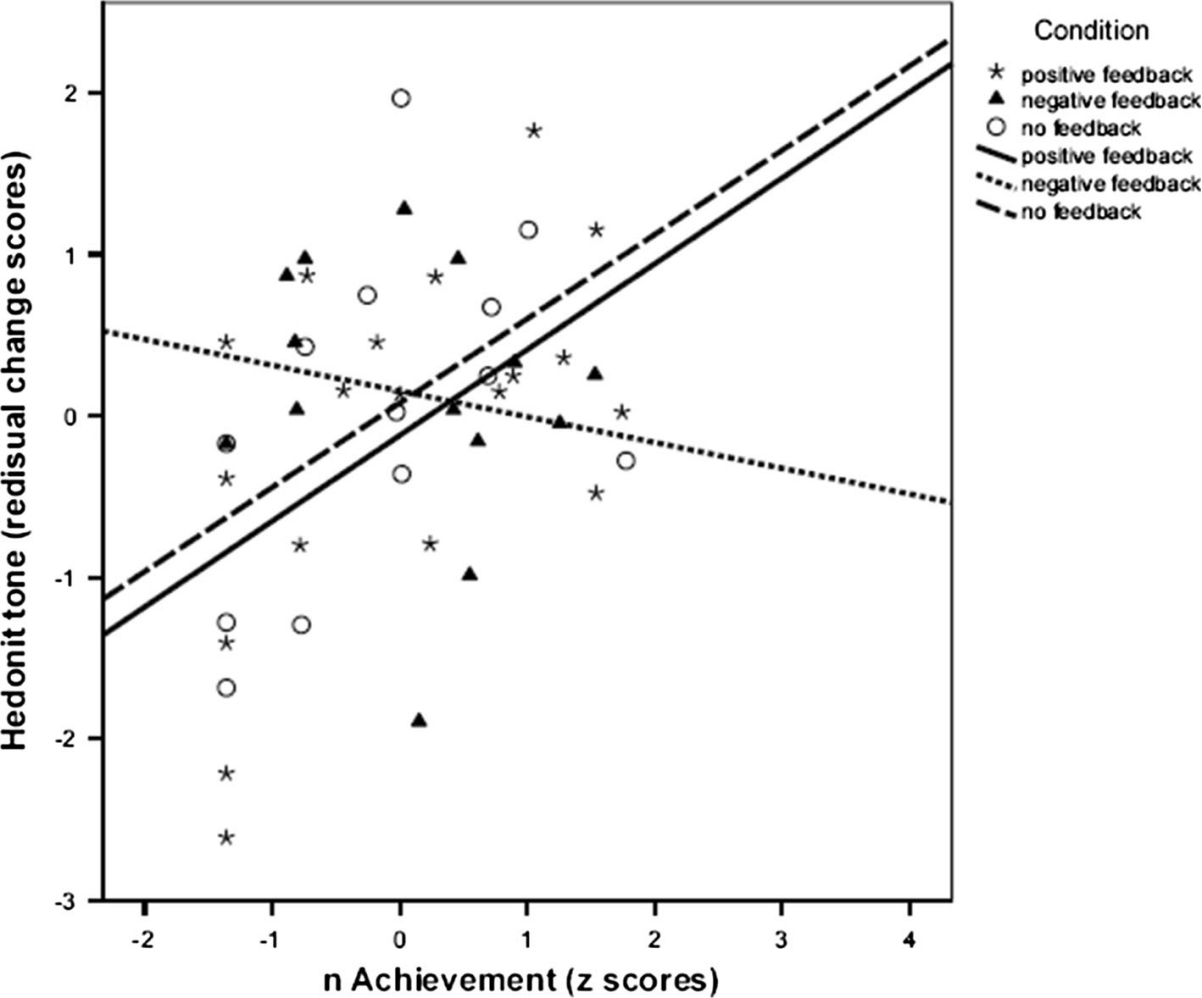
Relationship between *n*Ach and residualized hedonic tone scores in the three experimental conditions. (N = 46)

Regarding the correlations between *n*Ach and residualized post-task cortisol and felt affect in the whole sample and in each condition separately, we found similar results except that *n*Ach was positively related to residualized post-task cortisol in the whole sample (*r* = .44, *p* = .002), and *n*Ach was not significantly related to residualized post-task cortisol in the negative feedback condition (*r* = −.24, *ns*; Table 5). These results are illustrated in Figs. 4, 5.

**Table 5:** Bivariate correlation between Z score of *n*Ach and residualized outcomes (*N* = 46)

Condition		Residualized cortisol	Residualized EA	Residualized TA	Residualized HT
The whole sample	*n*Ach	0.44^**^	0.22	−.36^*^	.37^*^
Positive feedback (N = 19)	*n*Ach	.66^**^	0.21	−0.31	.54^*^
Negative feedback (N = 14)	*n*Ach	−0.24	0.06	−0.38	−0.17
No feedback (N = 13)	*n*Ach	.54^+^	0.30	−0.52	0.50

*nAch* need for achievement, *EA* energetic arousal, *TA* tense arousal, *HT* hedonic tone

** *p* < .01; * *p* < .05; + *p* < .07

Comparing the results including Participant A to those excluding Participant A, we found that the two sets of results differ only in terms of degree, rather than in terms of quality. For instance, we found significant interaction between *n*Ach and negative feedback with no feedback condition as the reference category for post-task cortisol under both of the two conditions. Post-hoc analyses show that *n*Ach was not significantly related to residualized post-task cortisol in the negative feedback condition (*r* = −.24, *ns*; *N* = 46), and *n*Ach was only marginally significantly related to residualized post-task cortisol in the negative feedback condition (*r* = −.50,*p* = .061; *N* = 47). Importantly, as follow-up simple slopes analyses show, *n*Ach is positively related to cortisol reactivity (compare Figs. 2 and 4) and to hedonic tone (compare Figs. 3 and 5) for the negative feedback condition, whereas the slopes in the other two conditions are all positive—regardless of whether or not Participant A’s data are excluded. As such, the direction and the gist of the findings for the two conditions are similar.

## Discussion

Our study examined whether *n*Ach moderates the relationship between achievement-related challenge and HPA activation, and also explored whether this relationship is influenced by the type of performance feedback participants received. We tested the hypothesis that individuals high in *n*Ach tend to have a lower cortisol response to a cognitively challenging task than those low in *n*Ach. The results showed that high *n*Ach was associated with a dampened cortisol response to the challenging task, but only when participants received negative feedback. On the other hand, when participants received positive feedback, high *n*Ach predicted a higher cortisol response to the challenging task, and participants in the no feedback condition showed a similar trend. Thus, the study only partially supported the hypothesis. Furthermore, our results indicate that the changes in cortisol were accompanied by changes in felt affect—specifically an increase in hedonic tone. Together, these findings build on the work of Wirth et al. (2006), demonstrating that important individual difference variables, such as implicit motives, can moderate the effect of motive-relevant stress on HPA activity.

Generally, these results speak of an enhanced cortisol response in the positive and no feedback condition, but a decreased cortisol response in the negative feedback condition. Our results in the negative feedback condition replicate the cortisol-damping effect of *n*Ach observed by Schultheiss et al. (2014). When engaged in a cognitively challenging task, participants high in *n*Ach may perceive the negative feedback as a sign of greater opportunities to experience mastery, thus leading to a lower cortisol response than those lower in *n*Ach. This is consistent with the results of Brunstein and Gollwitzer (1996), who found that individuals who received negative feedback on their performance in the d2 test reported the task to be more engaging than those who were not given feedback. These findings are also in line with Wirth et al. (2006) research that showed that cortisol levels decrease during dominance contests, but only for those individuals who are high in power motive.

Our finding that positive feedback has similar effects to no-feedback on cortisol response is interesting to some extent unexpected. One possible explanation is that the task in the no- and positive-feedback conditions was not deemed to be sufficiently difficult or challenging for participants high in *n*Ach to fulfill their need to pursue accomplishment, thus leading to an increased cortisol response. However, there is also an alternative explanation: that the rise in cortisol in the positive feedback condition is a physiological indication of a positive response to the achievement challenge. There is some evidence linking moderately increased cortisol to a host of adaptive coping responses to stressors, such as increasing active coping and selective attention (van Honk et al. 2000). Perhaps the higher cortisol response in *n*Ach-motivated individuals in the positive feedback condition is reflective of such an active adaptive response. Nonetheless, this explanation is unlikely because of the highly similar cortisol response in the no-feedback condition.

Moreover, our analyses indicated that changes in hedonic tone, and not energetic or tense arousal, accompanied the effects of achievement-related challenge on salivary cortisol in *n*Ach-motivated individuals. Research on implicit motives and emotional well-being suggests that higher levels of *n*Ach predict greater positive felt affect (i.e., hedonic tone) when people engage in achievement-relevant task and make good progress (see Brunstein 2010, for a review). Consistent with this view, engagement in an achievement task and even the receipt of positive feedback in the task should logically elicit affective changes in positive felt affect for individuals high in *n*Ach. The hedonic tone subscale of the UMACL measures precisely this construct, asking participants to rate how “cheerful”, “happy”, “depressed” (reverse scored), or “gloomy” (reverse scored) they felt.

Finally, we observed an unexpected negative relationship between *n*Ach and changes in tense arousal across all feedback conditions, which may be indicative of perceived threat among individuals not motivationally-oriented towards such a task. Schultheiss and Brunstein (2005) argue that task difficulty is a deterrent for those low in *n*Ach, many of whom will have learned to shun achievement-related situations after previously being punished for engagement in achievement activity. Given that low *n*Ach individuals are in a chronic state of passive avoidance manifesting as a desire to avoid achievement situations, participation in achievement tasks may be perceived as threatening, an aversive state that is likely to increase endorsement of items on the tense arousal subscale of the UMACL (e.g. “anxious”, “nervous”, “tense”). If so, this would explain the significant negative relationship between *n*Ach and tense arousal observed in both the correlational analysis and the ANCOVA.

Several limitations in the present study should be noted. First, cortisol was only measured twice and over a relatively wide time window spanning the late morning to early afternoon, although the placement of the post-task sample was close to the 20-min post-event maximum of the cortisol response identified by Dickerson and Kemeny (2004) in a meta-analysis. But more frequent post-task sampling might have helped to better home in on *n*Ach-dependent maxima in cortisol release, and subsequent research should therefore measure cortisol at more time points in the afternoon in order to more accurately capture the trajectory of cortisol change and to minimize cortisol variations due to circadian rhythmicity. In addition, there might be variations of cortisol reactivity in different menstrual cycle phases and future research would benefit from controlling for the effect of cycle. Second, the study would have benefitted from an additional non-performance control condition in which participants did not engage in the achievement task, as this would have allowed comparison of the cortisol response of high and low-*n*Ach individuals under both challenging and non-challenging conditions. Third, with only 47 participants, the present study had low power to detect reliable effects. We note however, that despite this shortcoming, the negative-feedback condition replicated the cortisol-damping effect of *n*Ach observed by Schultheiss et al. (2014). Fourth, in our research, we only examine one arm of the stress response (HPA axis) and ignore the other one (SNS). Since the meaning of the overall stress response can only be gauged by looking at both at once, we recommend that future replications should include candidate measures of SNS activity (such as salivary alpha-amylase or sAA; see Nater and Rohleder 2009). Finally, we did not conduct a manipulation check in order to confirm that the participants found the cognitive task to be sufficiently challenging. Specifically, it would be helpful to see whether participants in the negative feedback condition did perceive the achievement task as more challenging than participants in the other conditions. Future replications and extensions should attempt to address these concerns.

## Conclusion

While previous studies have emphasized the role of the HPA axis in responding to threat, our results are consistent with a general role in responding to non-social-evaluative challenge as well. While some challenges (such as social evaluations) may be universal, others may be more or less relevant for different individuals, manifesting as varied responses to challenge in different domains. In the present study, we demonstrated that individual differences in achievement motive moderate the effect of motive-relevant challenge on cortisol reactivity, and that the changes in cortisol while engaging in a motive-relevant task are accompanied by changes in felt affect in the same direction, specifically hedonic tone.
